# Silicification in Grasses: Variation between Different Cell Types

**DOI:** 10.3389/fpls.2017.00438

**Published:** 2017-03-28

**Authors:** Santosh Kumar, Milan Soukup, Rivka Elbaum

**Affiliations:** Robert H. Smith Institute of Plant Sciences and Genetics in Agriculture The Hebrew University of JerusalemRehovot, Israel

**Keywords:** cell wall, grasses, inflorescence bracts, root endodermis, silica cells, silicification mechanism, transpiration, trichomes

## Abstract

Plants take up silicon as mono-silicic acid, which is released to soil by the weathering of silicate minerals. Silicic acid can be taken up by plant roots passively or actively, and later it is deposited in its polymerized form as amorphous hydrated silica. Major silica depositions in grasses occur in root endodermis, leaf epidermal cells, and outer epidermal cells of inflorescence bracts. Debates are rife about the mechanism of silica deposition, and two contrasting scenarios are often proposed to explain it. According to the passive mode of silicification, silica deposition is a result of silicic acid condensation due to dehydration, such as during transpirational loss of water from the aboveground organs. In general, silicification and transpiration are positively correlated, and continued silicification is sometimes observed after cell and tissue maturity. The other mode of silicification proposes the involvement of some biological factors, and is based on observations that silicification is not necessarily coupled with transpiration. Here, we review evidence for both mechanisms of silicification, and propose that the deposition mechanism is specific to the cell type. Considering all the cell types together, our conclusion is that grass silica deposition can be divided into three modes: spontaneous cell wall silicification, directed cell wall silicification, and directed paramural silicification in silica cells.

## Introduction

Silicon is a ubiquitous soil element that along with oxygen forms 50–70% of soil mass (Ma and Yamaji, 2006). Plant roots absorb silicon as mono-silicic acid [Si(OH)_4_], a solute that is released to soil by the weathering of siliceous minerals. Near most soil pH, silicic acid is an uncharged molecule with pKa 9.8. Its concentration in soil solutions usually varies between 0.1 to 0.6 mM, but may range anywhere between 0.01 to 2.0 mM (Haynes, 2014). Silicon affects plants’ physiology in many beneficial ways, imparting tolerance against biotic stresses and alleviating adverse effects of abiotic stresses (Liang et al., 2015). Although the benefits of silicon in agriculture are known for a long time, no general mechanism of action was defined.

Silicon content across plants varies between about 0.1% to more than 10% on dry weight basis (Epstein, 1994). Plants belonging to fern family Equisetaceae, and monocots belonging to families Poaceae, Cyperaceae, and Commelinaceae have relatively large silicon content of about 4% (Hodson et al., 2005; Currie and Perry, 2007). Among them, Poaceae (the grass family) is the agriculturally most important family, with rice, wheat, and barley constituting the basis for human nutrition worldwide. In grasses, the root uptake of silicic acid (herein referred to as Si) is mediated by the cooperative action of an aquaporin-like channel Low silicon 1 (Lsi1) and a proton antiporter, Lsi2. Afterwards, Si moves with the transpiration stream and is unloaded from the xylem in the leaves by another aquaporin-like channel, Lsi6 (reviewed by Ma et al., 2011; Ma and Yamaji, 2015). In addition, so far unknown Si transporters might be involved in directing further Si transfer within the leaf tissues and concentrating it in target locations. The solute terminally polymerizes with concomitant loss of water molecules, forming hydrated silica (SiO_2_⋅nH_2_O). Plant silicification occurs in cell walls, cell lumens, and intercellular spaces. While most of the mineral is found in the shoot, some Si polymerizes in the roots (Sangster, 1978).

Two contrasting hypotheses are often proposed to explain silica deposition. The first is based on a passive mode of silicification, relying on the spatial correlation between silica deposition and organ transpiration (Yoshida et al., 1962; Sangster and Parry, 1971; Rosen and Weiner, 1994; Euliss et al., 2005). In this case, specific cell wall components and cuticular structures may additionally affect the location of bio-silicification (reviewed by Exley, 2015; Guerriero et al., 2016). This hypothesis infers that silica deposition in plants is a spontaneous process resulting from auto-condensation of Si molecules as the sap undergoes dehydration (Yoshida et al., 1962). The second hypothesis suggests that the formation of plant silica structures is catalyzed by biological entities (Kaufman et al., 1969; Hayward and Parry, 1973; Sangster et al., 1983; Parry et al., 1984; Kumar et al., 2017). Some authors suggest that silica deposition cannot be explained solely by any one of the two hypotheses, and both the mechanisms may be involved simultaneously (Hayward and Parry, 1975; Sangster et al., 2001; Motomura et al., 2004; Markovich et al., 2015). A review of literature is thus pertinent to better understand this biomineralization process.

## Sites of Silicification in Grasses

**FIGURE 1:**
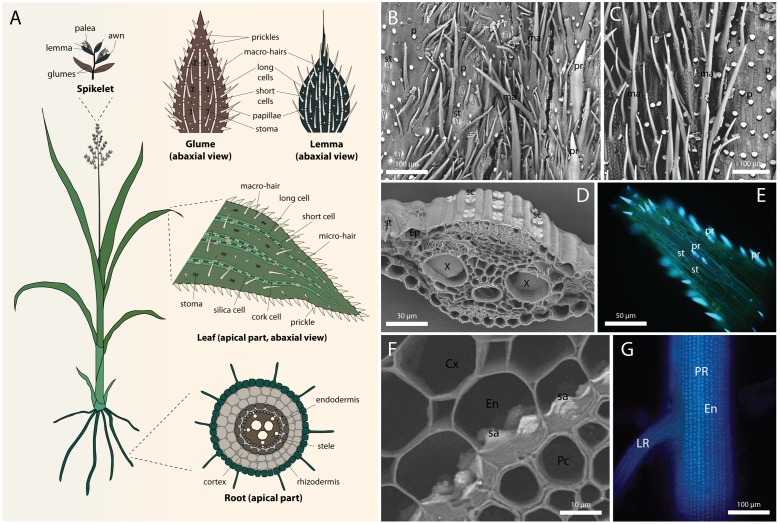
**Silica deposition in grasses.** **(A)** Diagram showing a full view of a generalized grass, and typical silicification patterns in the inflorescence (top), leaf epidermis (middle), and root cross-section (bottom). White represent silicified cells. **(B)** Scanning electron micrograph (SEM) of the abaxial epidermis of glume in *Triticum aestivum* L. **(C)** SEM of the abaxial epidermis of lemma in *T. aestivum*. **(D)** SEM of *Sorghum bicolor* (L.) Moench leaf cross-section showing silica cells in the epidermis. **(E)** Fluorescence micrograph of prickles at the leaf tip in *S. bicolor* visualized by alkali-induced fluorescence (Soukup et al., 2014). **(F)** SEM of *S. bicolor* root cross-section showing silica aggregates anchored in the inner tangential cell walls of endodermis. **(G)** Alkali-induced fluorescence micrograph of *S. bicolor* primary root showing extensive distribution of silica aggregates in the endodermis. Root cortex was mechanically removed to expose the inner tangential cell walls. Cx, cortex; En, endodermis; Ep, epidermis; LR, lateral root; ma, macro-hair; p, papilla; Pc, pericycle; PR, primary root; pr, prickle cell; sa, silica aggregate; sc, silica cell; st, stoma. SEMs were collected at the back scattered electron mode, rendering silicon atoms brighter than carbon atoms.

Silica is deposited in all the organs of grasses. The most intensely silicified tissues are usually root endodermis, leaf epidermis, and abaxial epidermis of inflorescence bracts (**Figure 1**). In most cases, silica impregnates the cell walls, directly laid down onto the cell wall matrix (Bauer et al., 2011; He et al., 2013; Hodson, 2016). The composition of the silicifying matrix may vary between species and cell types, thus influencing silicification pattern (reviewed by Guerriero et al., 2016; Hodson, 2016). In particular, grasses have a unique hemicellulose composition, containing glucuronoarabinoxylan and mixed-linkage glucans, instead of the xyloglucan in non-commelinid monocots and dicots. Furthermore, grass cell walls contain more phenylpropanoids and less pectin compared with dicots (Guerriero et al., 2016). Silica is often proposed to crosslink the cell wall polymers, adding to their compressive strength (Currie and Perry, 2009; He et al., 2013; Kido et al., 2015), similar to the role of lignin in lignified walls (Salmén, 2015). In addition, structural trade-off between silica, lignin and cellulose was observed in rice (Suzuki et al., 2012; Yamamoto et al., 2012) and in a number of wetland species (Schoelynck et al., 2010). As the metabolic costs of silica deposition were estimated to be 20-fold lesser than that of lignification (Raven, 1983), silicification can present preferable solution for improving mechanical properties of plant tissues. However, silica seems not to provide water repelling properties comparable to lignin and its utilization thus require some degree of regulation (Soukup et al., 2017).

### Silicification in Inflorescence Bracts

Translocation of Si in plants is driven mostly by transpiration (Sangster and Parry, 1971), and coordinated by specific distribution of Si transporters. Silicic acid is selectively transported to the panicle of rice during its maturation (Yamaji et al., 2015), possibly due to its increasing sink strength (Detmann et al., 2013). In panicles, Si is concentrated and deposited in the inflorescence bracts (Hodson and Sangster, 1988), which serve as a tough protecting shield to the developing caryopses. Silica deposition is restricted to the epicarp hairs (Bennett and Parry, 1981; Parry et al., 1984) and the outer wall of aleurone layer (Hodson and Parry, 1982), whereas it does not accumulate in the caryopsis endosperm (Jones et al., 1963). Bract silicification also provides a safe disposing location for the mineral, which would polymerize anyway, as the transpired water evaporates.

#### Silicification of the Glume Prickle Hairs, Papillae, and Long Cells

The abaxial epidermis of the *Phalaris canariensis* glume consists of stomatal complexes, long cells, prickle hairs, papillae, marco-hairs and silica-cork cell pairs (Hodson et al., 1985). **Figure 1B** shows a scanning electron micrograph of a *Triticum aestivum* (wheat) glume, exhibiting stomata, prickle hairs, papillae, and macro-hairs. In this section we will discuss silicification in glume prickle hairs, papillae and long cells, whereas the silicification of silica cell will be discussed in a separate section. Presence of stomatal complexes on the abaxial epidermis indicates substantial transpiration after their emergence. Nevertheless, the outer tangential cell wall of papillae and prickle hair tips are already silicified at emergence (Sangster et al., 1983; Hodson et al., 1985). The flag leaf sheath encloses the inflorescence before emergence and limits its transpiration, raising the possibility that the cell wall is conducive for spontaneous silica deposition (Nissan et al., 2015). Callose [β-(1→3)-D-polyglucose] induces silica deposition in undersaturated silicic acid solution (Law and Exley, 2011), and it is possible that similarly, other polysaccharides might play such role in cell wall silicification. Cross-sections of mature prickle hairs and papillae show that the lumen of these cells is also filled with silica, without the in-growth of cell wall into the cell lumen to template the silicification (Hodson et al., 1985; Hodson, 2016). Lumen silicification in dead cells suggests that it might be driven either by passive transpiration via silica granule formation (Bennett, 1982), or templated by organic matrix that is accessible to Si only after cell death (Sangster, 1970). However, the fact that lumen silicification continues long after cell death rather supports the transpiration driven passive mode of silicification. Thus, it might be possible that silica is deposited in two stages in these cells, starting at the tip and the outer wall induced by wall materials, followed by a spontaneous precipitation inside the lumen driven by the degradation of the protoplast and evapo-transpiration (Motomura et al., 2006; Markovich et al., 2015).

In long cells, the outer wall thickens about 1 week after glume emergence. Silica deposition seems to initiate 2 weeks after the glume emergence, in parallel to cell death and collapse of the long cells and parenchymatic cells (Hodson et al., 1985). Thus, long cell silicification seems to depend on water evaporation.

#### The Outer Epidermal Cells and Macro-Hairs in Lemma

The outer epidermis of the lemma of *Phalaris canariensis* lacks stomata, suggesting low transpiration rates (Tambussi et al., 2007). Silicification, however, occurs in macro-hairs and typical rectangular epidermal cells (Hodson et al., 1984), a trait common to many grasses. Before panicle emergence, both the macro-hairs and outer epidermal cells have a large vacuole. The presence of Si was not detected at this stage. **Figure 1C** shows a scanning electron micrograph of a *T. aestivum* glume, exhibiting papillae, and macro-hairs, but no stomata.

Macro-hairs are unicellular trichomes, often with lengths greater than 1 mm on *Phalaris canariensis* lemma (Perry et al., 1984a). Macro-hairs also start to silicify after panicle emergence. Silica deposition initiates at the hair tip (Perry et al., 1984b). During the week following emergence, wall thickening proceeds to the base, and the outer layer of the wall is silicified. The nanometric morphology of the deposited silica is governed by the newly laid-down polysaccharides. Sheet-like structures form during the deposition of arabinosylated xylan and cellulose, globular particles deposit simultaneously with mixed linkage β-(1→3, 1→4)-D-glucan, and fibrous silica forms after the cell wall thickening stops. Apparently, the polysaccharides provide chemical environments necessary to stabilize the deposited silica (Perry et al., 1987). By 2 weeks after emergence, silicification of the wall material continues in concentric rings (Hodson et al., 1984; Perry et al., 1984a). The cytoplasmic content of macro-hairs breaks down leaving behind an empty lumen (Hodson et al., 1984).

Deposition of silica at the outer epidermal cells is also templated by their cell wall. At emergence, the inner tangential cell walls thicken to occupy most of the cell volume, leaving only little space for active cytoplasm. In the week following emergence, cytoplasm degrades and silicification initiates in the cell wall region close to the pre-existing cytoplasm. Within 2 weeks, the whole cell wall is impregnated with silica (Hodson et al., 1984). We conclude that in macro-hairs and outer epidermal cells of the lemma, silicification templated by the cell wall coincides with the onset of spikelet transpiration.

### Leaf Silicification

Among all plant organs, leaves usually exhibit highest transpiration volumes. The evaporation of water promotes xylem sap condensation and contributes to the formation of solute sediments, including silica. However, even though most of the water evaporates from mesophyll cells and passes through stomata to the atmosphere, silicification of the guard cells occurs at slow pace, advances with age, and never reaches all of the cells (**Figures 1D,E**), (Motomura et al., 2004). Among other epidermal cell types, long cells accumulate silica in their walls as soon as the leaf starts to transpire (Sakai and Sanford, 1984). Silicification in the mesophyll and bulliform cell walls is rather characteristic to mature, sometimes senescent leaves (Sangster, 1970; Dinsdale et al., 1979). Silicification is probably spontaneous in the cell wall of these cells, resulting from Si auto-polymerization. In case of lumen silicification in bulliform cells, granules of silica are observed (Motomura et al., 2004).

#### Silicification of Leaf Micro-Hairs

**FIGURE 2:**
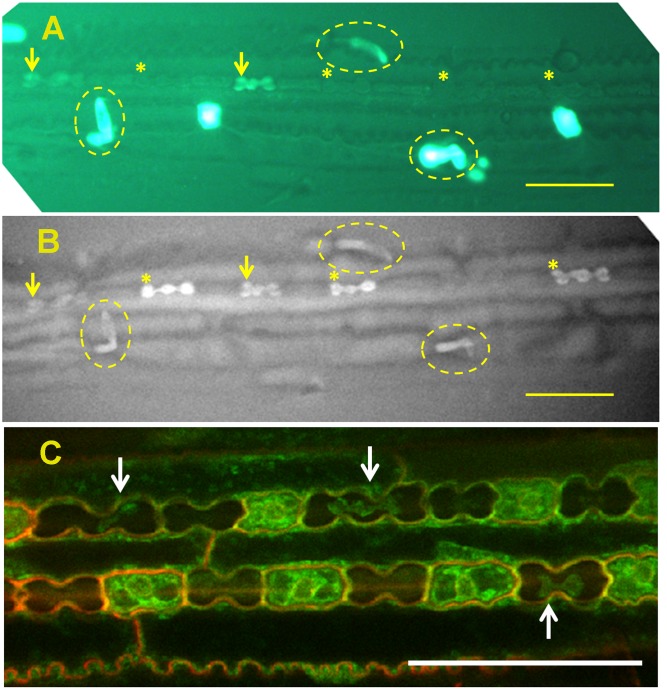
**Silica deposition in the epidermis of sorghum leaf.** **(A)** Viability assay of epidermal peel showing viable cells’ cytoplasm green. Viable silica cells are indicated with arrows whereas dead silica cells are indicated with asterisks. Micro-hairs are shown with broken ovals. **(B)** Back-scattered electron micrograph of the same field of view, showing high signal intensity emanating from viable silica cells (arrow) and micro-hairs (broken oval). Dead silica cells are already silicified (asterisks), although one dead non-silicified silica cell can also be seen [compare **(A,B)**]. **(C)** Silica cells displaying shrunken but viable cytoplasm (arrows) indicating extra-membranous silica deposition. All scale bars represent 50 μm. Images adapted from Kumar et al., (2017) with permission from the John Wiley and Sons publications. Copyrights of the image rest with the original authors and publisher.

Micro-hairs are bicellular trichomes having a basal and a cap cell. Basal cells of micro-hairs in sugarcane are the first cell type to silicify, even before leaf exposure to the outside environment (Sakai and Sanford, 1984). In sorghum, silicification of the basal cell initiates in viable cells (**Figure 2**), probably at the cell wall. The cells probably die later on, and their lumen passively fills up with silica (Motomura et al., 2000). In bamboo (*Sasa veitchii*), cap cells accumulate significant amounts of silica only after leaf opening. The number of silicified micro-hairs increases with age (Motomura et al., 2004), suggesting a dependency on transpiration. Silicification in micro-hairs seems thus to share similar mechanism to prickle hairs and papillae in the inflorescence bracts.

#### Silica Deposition in Silica Cells

In grasses, silica cells are found as stretches of silica-cork cell pairs in the epidermis of leaves (Sangster, 1970), stem internodes (Kaufman et al., 1969) and abaxial epidermis of glumes (Hodson et al., 1985). Silica cells are among the first type of cells to be silicified in a tissue, sometimes even before the tissue is exposed to the atmosphere (Kaufman et al., 1969; Sangster, 1970; Motomura et al., 2006; Kumar et al., 2017). The silica deposition occurs over hours (Blackman, 1969; Kaufman et al., 1969), suggesting that the process is metabolically controlled. In rice, the walls of silica cells lignify before silica deposition (Zhang et al., 2013). Prior to silica deposition, silica cells are metabolically very active with large nucleus and high numbers of ribosomes and mitochondria. Silica cells are well connected to their neighboring cork cells, but not to the neighboring long cells (Lawton, 1980). Silica cells are viable at the time of silica deposition. The mineralization process initiates in the extra-membranous space and proceeds centripetally (**Figure 2**). The forming mineral is limited by the cell wall on one side and by the membrane on the other side. The shrunk cytoplasm maintains its viability and the deposited silica does not interfere with cell-to-cell diffusion (Kumar et al., 2017). Silica deposition occurs also in leaf segments with very limited transpiration flow (Sangster and Parry, 1971; Kumar et al., 2017). The deposition is reduced in leaves treated with a metabolic inhibitor 2,4-dinitrophenol (Sangster and Parry, 1971) and does not occur in dead silica cells present in live leaves (Markovich et al., 2015; Kumar et al., 2017). These findings further indicate on a metabolic process controlling the silicification.

Organic matter with N/C ratio indicative of amino acids is continuously distributed in wheat silica cells (Alexandre et al., 2015), suggesting possible role of proteins in the templating process, similar to diatoms (Kröger et al., 1999). Depending upon the phytolith extraction process, some of nitrogen detected may result from the use of nitric acid in the extraction process or represent acid hydrolyzed proteins occluded within the silica structure (Watling et al., 2011), even without their direct participation in silicification.

### Silicification in Roots

Roots are the first organs exposed to silicic acid, allowing its uptake and controlling the extent of Si supply to the entire plant. In most cases, the deposition of silica in roots cannot rely on evaporation of water for concentrating silicic acid. A passive mode of silicification in roots thus assumes that Si condensation occurs as water is absorbed by the symplasm, leaving behind concentrated silicic acid solution in the apoplasm (Exley, 2015). Such separation may occur at the Casparian bands, where the passive diffusion of Si and water is blocked (Sakurai et al., 2015). Indeed, in the aerial parts of adventitious roots of *Phalaris canariensis*, silica deposition occurs in the epidermis and outer cortical layer, but not in the endodermis (Hodson, 1986). This observation suggests the involvement of transpiration in silica deposition in these aerial roots. However, transpiration-dependent model does not conform to silicification at the endodermal inner tangential walls, which are located centripetally to the Casparian bands (Sangster, 1978). Depending on the grass species, silica impregnates the endodermal inner tangential and radial cell walls (Parry and Soni, 1972; Hodson and Sangster, 1989; Lux et al., 2003a), or forms discrete aggregates anchored in the inner tangential wall (Sangster and Parry, 1976a; Parry and Kelso, 1977; Sangster, 1977) (**Figures 1F,G**).

The active uptake of Si that bypasses the Casparian bands occurs at the apical part of roots. Afterward, Si is transferred with the transpiration stream basipetally through the central cylinder (Lux et al., 2003b; Soukup et al., 2017). While most of the Si is transported to the shoot, some Si binds to the root endodermis with high affinity (Markovich et al., 2015). Endodermal silicification is usually associated with the thickening of inner tangential walls and the deposition of polyphenols suberin and lignin in the mature parts of the roots (Parry and Kelso, 1975; Sangster and Parry, 1976a). Since Si is taken up actively in the root apex, the mature endodermis is supplied with Si by its centrifugal flow from the central cylinder. This model was evidenced in sorghum, where the aerial parts of adventitious roots silicified even before reaching the growth medium (Sangster and Parry, 1976b). Accordingly, silica deposition was detected in the basal parts of roots grown with Si supply provided to their apices only (Lux et al., 2003b), even if the cortical tissues between those regions were removed (Soukup et al., 2017).

In sorghum, silica aggregation initiates in non-lignified sites of the inner tangential cell walls, possibly templated by arabinoxylan–ferulic acid complexes. The aggregation sites are established even in the absence of Si, indicating that the formation of silica aggregates is at least partially controlled by the structure and composition of the endodermal cell walls (Soukup et al., 2017). The aggregates seem to swell the silicifying wall and protrude from the endodermal inner tangential wall toward the cell lumen (**Figure 1F**).

In older roots, the deposition of silica can extend also to other root regions, e.g., to the outer tangential walls of endodermis or intercellular spaces of cortex. Such deposition is probably a result of Si condensation due to water uptake by the symplasm, water evaporation, or it can be induced by increasing ionic strength of the apoplasm or pH changes. With the increasing age of the roots, stele, sclerenchyma and conductive tissues may also silicify (Parry and Kelso, 1975; Montgomery and Parry, 1979; Hodson and Sangster, 1989).

## Conclusion

Transpiration plays a major role in moving Si throughout the plant. Water evaporation and water uptake by the symplasm can efficiently condense Si and lead to silica precipitation. However, uncontrolled and spontaneous silica deposition may be harmful for the functioning of the plant. The evolution of mechanisms for a safe disposal of Si was thus essential. Based on the matrix that templates the silicification and the participation of transpiration in this process, we identified three types of silica deposition in grasses that describe silicification in most of the cell types. (1) Passive cell wall silicification: This type is distinctive to mature and/or intensely transpiring organs, where the condensation of Si is driven by dehydration. A continuous supply of Si infiltrates the non-silicified cell walls and its deposition occurs without being metabolically controlled by the cells. (2) Controlled cell wall silicification: Silica is deposited directly on the cell wall matrix, even before the organ is exposed to the atmosphere/transpiration. Silicification is possibly templated by the cell wall polymers inducing the silicic acid polymerization. In some cases, the cell protoplast dies, allowing spontaneous silica deposition driven by transpiration in the cell lumen, without further organic template. (3) Silica cells are a special case, where the mineral is deposited on the external side of a functional plasma membrane, possibly in a volume that contains materials that enhance silica deposition, independent of transpiration.

Thus we saw, a plant as a whole does not follow one silicification mechanism but the observed mechanism is specific to the cell-type chosen for study. Sometimes, a cell type follows two stage silicification: the early stage being cell wall silicification followed by granular silica deposition in the dead lumen. Thus, silicification in grasses is not an entirely active or passive process, and its mechanism is cell-type specific.

## Author Contributions

SK prepared the initial draft. All authors commented, added and revised the manuscript and approved for publication.

## Conflict of Interest Statement

The authors declare that the research was conducted in the absence of any commercial or financial relationships that could be construed as a potential conflict of interest.
